# Immune normalization strategy against suboptimal health status: safe and efficacious therapy using mixed-natural killer cells

**DOI:** 10.18632/aging.203279

**Published:** 2021-08-30

**Authors:** Ying Li, ODA Harunori, Shihu Fu, Fuyuan Xing, Huawan Wu, Juan Wang, Aihua Chen, Xinhua Ren, Dawei Peng, Xia Ling, Ming Shi, Hongjin Wu

**Affiliations:** 1 International Research Center for Regenerative Medicine, BOAO International Hospital, Qionghai 571434, Hainan, China; 2 School of Life Science and Technology, Harbin Institute of Technology, Harbin 150001, Heilongjiang, China; 3 Medical Corporation ISHIN-KAI ODA Clinic, Shinjuku-ku 169-0072, Tokyo, Japan

**Keywords:** immune cell therapy, natural killer cells, CD16, CD56, PD-1

## Abstract

“Immune normalization” has emerged as a new paradigm in immunotherapy, which is proposed in cancer patients instead of conventional “immune-enhancement” therapy. Immune normalization may also be implemented in cancer prevention of “sub-healthy” individuals. We established *in vitro* cultured mixed-natural killer (NKM) cells to achieve immune normalization. The *in vitro* cytotoxicity of NKM cells was tenfold higher than that of peripheral blood mononuclear cells (PBMCs). The cytotoxicity of NKM cells was negatively correlated with the proportion of T-helper cells (cluster of differentiation: CD3+CD4+ T), and positively correlated with the proportion of NK cells (especially CD56^bright^CD16^bright^ NK cells). Then, we defined “sub-healthy individuals” after measuring Programmed cell death protein-1 (PD-1) expression in PBMCs from 95 donors aged > 50 years. Furthermore, we evaluated the potential clinical application of NKM-cell therapy in 11 patients with malignant lymphoma, one patient with pancreatic cancer, and four sub-healthy individuals. NKM-cell therapy elicited good tolerance and side-effects were not found. In sub-healthy individuals, the proportion of CD3^+^PD-1^+^ T cells and CD3^+^CD8^+^PD-1^+^ T cells was reduced significantly after NKM-cell treatment. We demonstrated that a new method using NKM cells was safe and efficacious as adjuvant treatment for cancer patients as well as therapy for sub-healthy individuals. Normalization of the peripheral immune system through NKM-cell therapy could expand its scope of application in different disorders.

## INTRODUCTION

The immune system is a crucial barrier for the human body to resist diseases. Its ability has rhythmic fluctuations [1, 2], and is affected by several factors (e.g., genetic, age [3, 4]). Reduction in the response capacity of immune cells can lead to diseases (e.g., cancer), whereas excessive activation of immune cells can cause autoimmune diseases. Thus, balance in the immune system is important for health [5].

Recently, the concept of “immune normalization” in cancer therapy was investigated by Chen and colleagues [6]. If individual immunity can be normalized, various diseases may be prevented. The development of cell therapy in the past decade has provided hope that this strategy may work. Cell therapies, especially those using immune cells, have been used for cancer treatment, such as therapy using natural killer (NK) cells [7] and chimeric antigen receptor T-cells [8].

In recent decades, epidemiological data for colorectal cancer and lung cancer have revealed that prevention and early detection of cancer are far more efficacious than their treatment for controlling cancer mortality [9, 10]. Application of immune-cell therapy in disease prevention (especially cancer) can greatly reduce the occurrence or relapse of diseases [11, 12]. For older individuals who may have long-term immunocompromise, immune-cell therapy could “rebalance” their immune system, which would eliminate mutant cells and pathogens (e.g., viruses, bacteria), thereby reducing the disease risk.

Expression of programmed cell death protein (PD)-1 on T cells has been considered a sign of “immune exhaustion” [13, 14]. PD-1 expression on peripheral-blood mononuclear cells (PBMCs), including cluster of differentiation (CD)4^+^ and CD8^+^ T cells, has been shown to be increased significantly in cancer [15–20], sarcoidosis [21] and chronic infection with the hepatitis-C virus [22]. In 2019, Cortese and colleagues found that in progressive multifocal leukoencephalopathy, PD-1 expression on PBMCs was high and could be reduced significantly after pembrolizumab treatment [23]. Furthermore, in patients suffering from severe coronavirus disease 2019 (COVID-19), in addition to a reduction in the number of peripheral-blood T cells [24–28], the proportion of PD-1 expression in the remaining T cells is very high [27, 28].

We established an *in vitro* cultured mixed-NK (NKM) cell system using PBMCs and then tested its potential anti-tumor activity *in vivo* and *in vitro*. Our data represent the first NKM-cell therapy, and could be used for healthcare and, possibly, cancer treatment.

## RESULTS

### Generation and characterization of NKM cells

**Figure 1:**
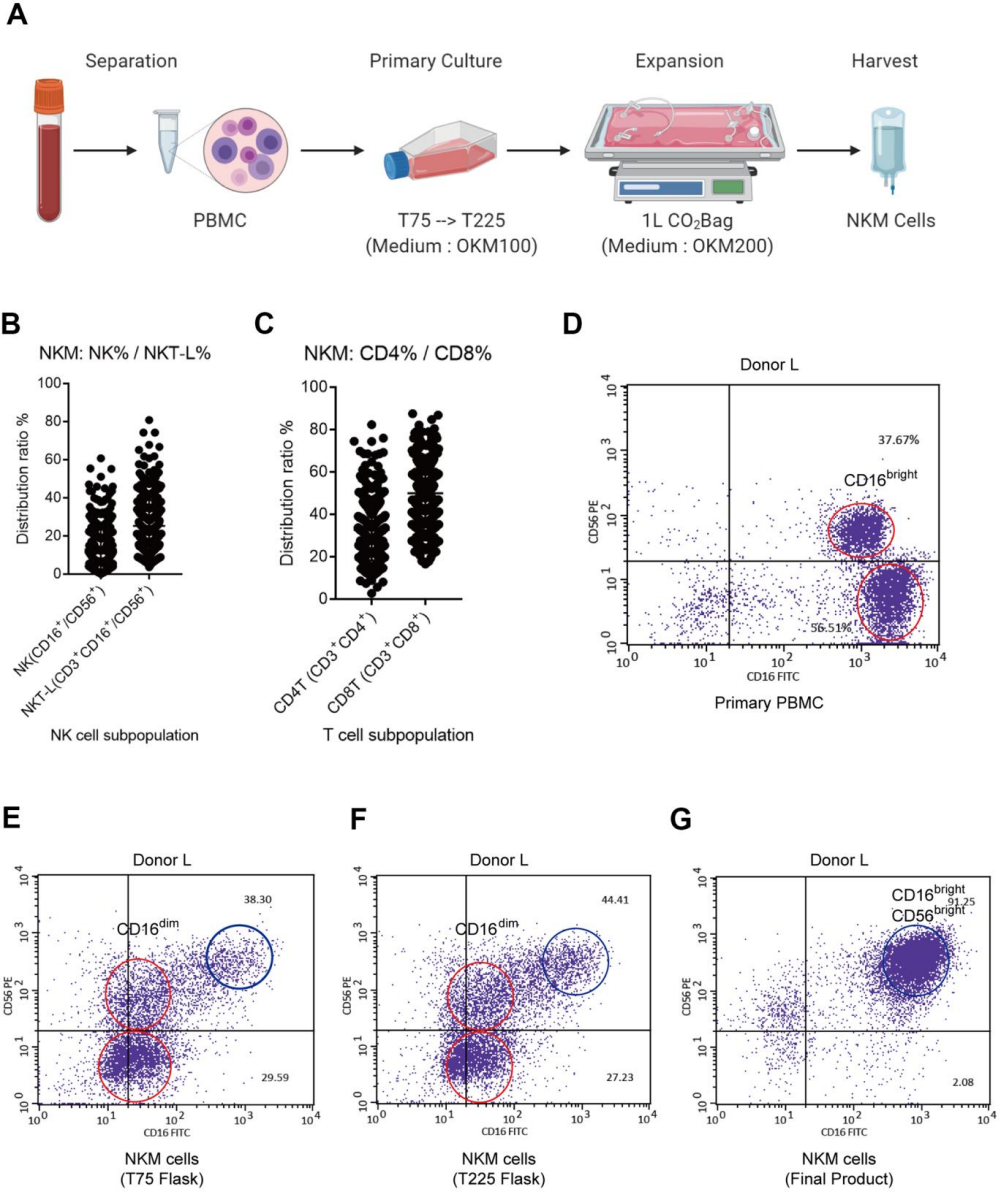
**Characterization of NKM cells.** (**A**) Manufacturing process of NKM cells. The whole process must be carried out in a GMP Laboratory Class A ultra-clean workbench. (**B**) Composition of NKM cells. (**C**) The main NK cells in NKM cells were CD16^bright^CD56^bright^ NK cells. The NKM cells from donor-N were analyzed by FACS. The main NKM cells were CD16^+^CD56^+^ cells (87.22%), whereas the majority was CD16^bright^CD56^bright^ NK cells. (**D**–**G**) NK subpopulations of NKM cells in different culture stages, including primary PBMCs (**D**), T75 flask (**E**), T225 flask (**F**), and NKM product (**G**). The red circle in primary PBMCs denotes CD16^bright^ NK cells, whereas the red circle in T75 and T225 flasks denote CD16^dim^ NK cells. The blue circle denotes CD56^bright^CD16^bright^ NK cells.

To achieve safe and efficacious immunotherapy for patients, we established an *in vitro* culture system for the production of immune cells without separation or biological additives. The medium used in this culture system was designed specifically for the expansion of NK cells and T cells. Autologous plasma was prepared and inactivated, then used for cell culture. Autologous immune cells were manufactured as shown in Figure 1A and in the Methods and Materials section: these were NKM cells. After the culture was complete, we could obtain ~2.5 billion immune cells, the main components of which were NK cells and T cells.

We analyzed the immune-cell subsets of NKM cells. The proportion of monocytes (CD14^+^), B cells (CD19^+^) and dendritic cells (CD11c^+^) was extremely low. In total, 369 NKM cells were analyzed for the distribution of NK cells (CD45^+^CD3^−^CD16/56^+^) and T cells (CD45^+^CD3^+^). NKM cells comprised ~20% NK cells (CD3^−^CD16/56^+^), ~30% NK T-like cells (CD3^+^CD16/56^+^), and other T cells. Of the T cells within NKM cells, ~30% were T helper (T_h_) cells (CD3^+^CD4^+^) and 50% were cytotoxic T cells (CD3^+^CD8^+^) (Figure 1B). In PBMCs, NK cells comprised mainly CD3^-^CD16^+^CD56^neg^ NK cells and CD3^−^CD16^+^CD56^dim^ NK cells (Supplementary Figure 1A). However, the NK cells from NKM cells mainly comprised CD3^−^CD16^+^CD56^+^ NK cells, of which the main subtype was CD3^−^CD16^bright^CD56^bright^ NK cells (Figure 1C and Supplementary Figure 1B). Furthermore, we analyzed the subpopulations of NK cells from donor-L at different stages of cell culture, including at the primary-culture stage (PBMCs, T75 flask, and T225 flask) and expansion stage (CO_2_ Bag). The main subpopulation of NK cells in PBMCs was CD16^bright^ NK cells (Figure 1D). The latter transformed into CD16^dim^ NK cells upon primary culture (Figure 1E, 1F), and finally became CD16^bright^CD56^bright^ NK cells after expanded proliferation (Figure 1G).

### *In vitro* cytotoxicity and its correlations with the subpopulation of NKM cells

**Figure 2:**
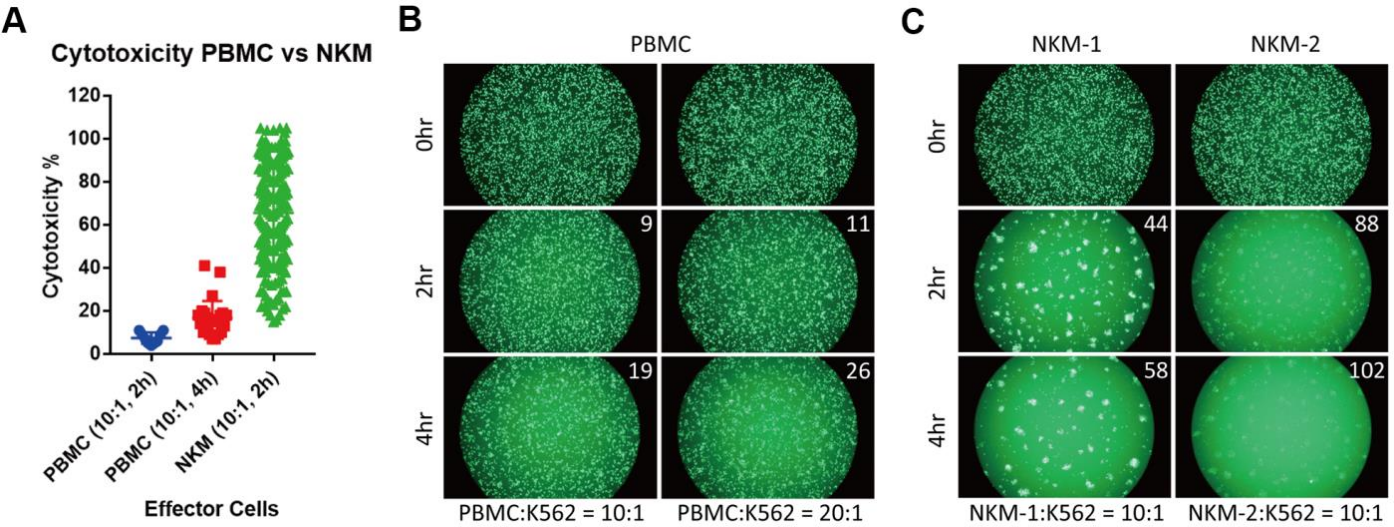
**NKM cells have very high *in vitro* cytotoxicity.** (**A**) The cytotoxicity of PBMCs and NKM cells. The cytotoxicity of 9 PBMCs (incubation for 2 h) and 28 PBMCs (incubation for 4 h) was obtained. The cytotoxicity of 198 NKM cells (incubation for 2 h) was obtained. (**B**, **C**) Labeled target cells (K562 cancer cells) were killed by effector cells (PBMC or NKM). The number on the upper right shows the cytotoxicity of effector cells. The ratio of effector cells: target cells was 10:1 or 20:1, and the incubation time was 2 h or 4 h.

We tested the *in vitro* cytotoxicity of NKM cells on a cancer cell line (K562), and compared its cytotoxicity with that of PBMCs and NK92 cells. In PBMCs (from healthy donors), the mean cytotoxicity was ~7.4% (effector/target (E/T) ratio = 10:1, 2-h incubation; n = 9) and 16.8% (E/T ratio = 10:1, 4-h incubation; n = 28). The mean cytotoxicity of NKM cells (n = 198) that we manufactured was ~65.6% (E/T ratio = 10:1, 2-h incubation), which was almost tenfold higher than that of PBMCs (Figure 2A and Supplementary Table 1). Approximately 84% of NKM cells showed >40% cytotoxicity. Even with more effector cells and longer incubation times, the cytotoxicity of PBMCs remained far inferior to that of NKM cells (Figure 2B, 2C). Then, the cytotoxicity of NK92 cells was investigated with different E/T ratios (Supplementary Figure 2A). We discovered that the cytotoxicity of NKM cells (E/T ratio = 10:1) was comparable with that of NK92 cells, with an E/T ratio of 5:1 (2-h incubation) (Supplementary Figure 2B). Taken together, these results suggested that, although NKM cells had only half the cytotoxicity of that observed in “pure” NK cells (NK92), their cytotoxicity was much higher than that of PBMCs.

**Figure 3:**
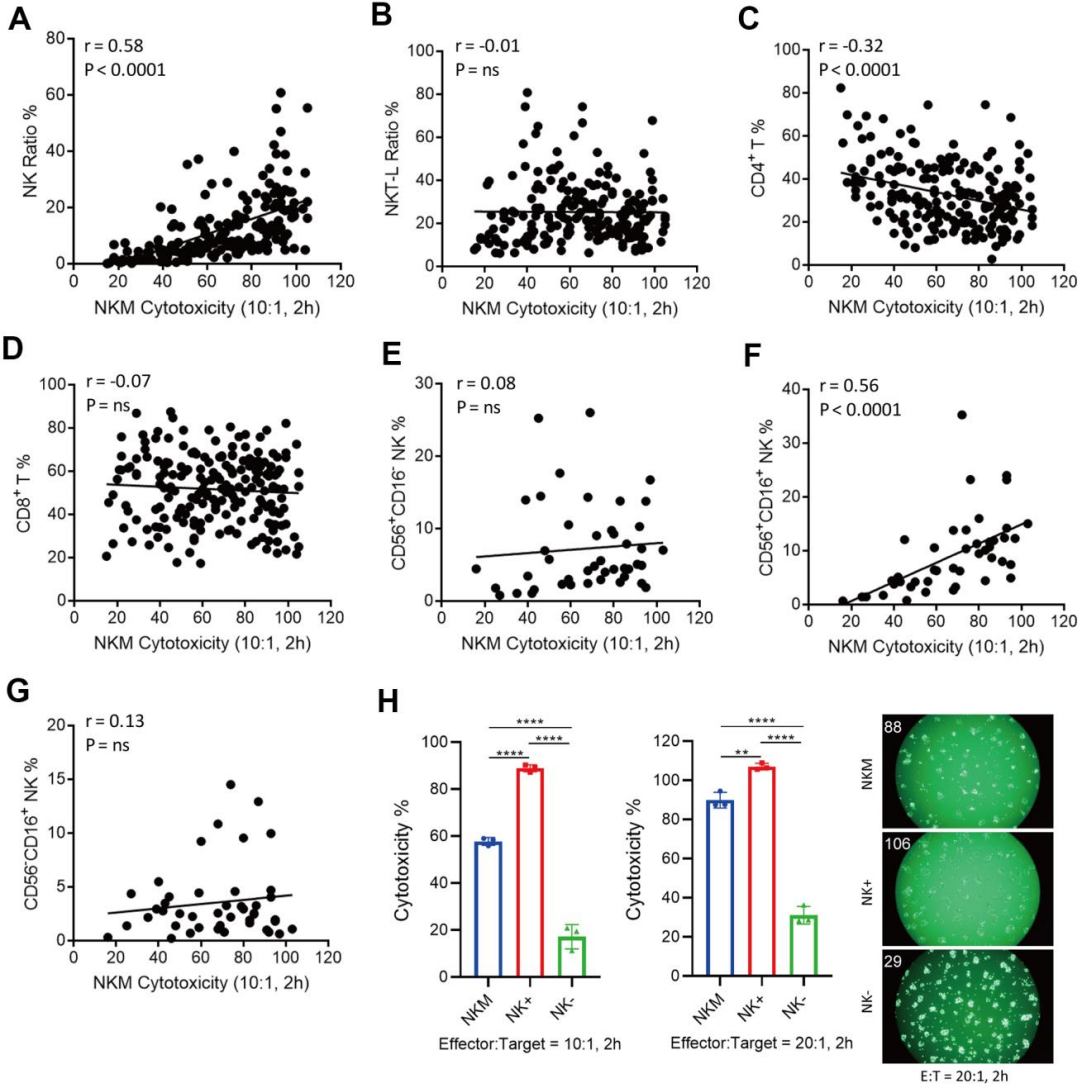
**Correlation of cytotoxicity of NKM cells and cell subpopulations.** (**A**–**D**) NK cells (CD3^−^CD16^+^/CD56^+^) were positively correlated with the cytotoxicity of NKM cells, whereas T-helper cells (CD3^+^CD4^+^ T) were negatively correlated with the cytotoxicity of NKM cells. A total of 198 NKM cells was used for correlation analyses, and four subpopulations were assessed: NK cells (**A**), NK T-like cells (**B**), CD3^+^CD4^+^ T cells (**C**) and CD3^+^CD8^+^ T cells (**D**). Correlations and linear regressions were analyzed with Prism 7.04 (GraphPad). (**E**–**G**) CD3^−^CD16^+^CD56^+^ NK cells were correlated the cytotoxicity of NKM cells. A total of 44 NKM cells was used for correlation analyses, and four subpopulations were evaluated: CD56^+^CD16^−^ NK cells (**E**), CD56^+^CD16^+^ NK cells (**F**) and CD56^−^CD16^+^ NK cells (**G**). Correlations and linear regressions were analyzed with Prism 7.04 (GraphPad). (**H**) The cytotoxicity of NKM, NK+ and NK- cells. The NKM cells were separated into NK+ (CD56^+^ or CD16^+^ NK cells) and NK- (other non-NK cells) cells with immunomagnetic beads (NK Cell Isolation Kit). The ratio of effector cells: target cells was 10:1 or 20:1, and the incubation time was 2 h. The number on the upper right shows the cytotoxicity of effector cells.

NKM cells are a mixed population of cells, so we wondered which subsets of these cells were responsible for *in vitro* cytotoxicity. We analyzed the subpopulations of NKM cells, and undertook correlation analyses of their *in vitro* cytotoxicity. We separated NKM cells into NK cells (CD3^−^CD16^+^ or CD56^+^), NK T-like cells (CD3^+^CD16^+^ or CD56^+^), CD4 T cells (T_h_ cells, CD3^+^CD4^+^) or CD8 T cells (cytotoxic T cells, CD3^+^CD8^+^). A strong positive correlation was observed between the cytotoxicity of NKM cells and the proportion of NK cells (r = 0.58, p < 0.0001), but a negative correlation with the proportion of T_h_ cells was noted (r = −0.32, p < 0.0001) (Figure 3A, 3C). There was no correlation with the proportion of NK T-like cells and cytotoxic T cells (Figure 3B, 3D).

Furthermore, we divided NK cells into three subpopulations: CD56^+^CD16^−^ NK cells, CD56^+^CD16^+^ NK cells, and CD56^-^CD16^+^ NK cells. The cytotoxicity of NKM cells showed a significant correlation only with CD56^+^CD16^+^ NK cells (r = 0.56, p < 0.0001), and no correlation was found in CD56^+^CD16^−^ NK cells or CD56−CD16^+^ NK cells (Figure 3E–3G). Then, we used sorting of immunomagnetic beads to separate different cell subpopulations in NKM cells, and verified them with flow cytometry. We discovered that NK cells isolated from NKM cells showed the highest cytotoxicity (Figure 3H), CD3^−^ cells, CD4^−^ cells and CD8^−^ cells showed moderate cytotoxicity, and NK-negative cells, CD3^+^ cells, CD4^+^ cells, and CD8^+^ cells showed the lowest cytotoxicity (Supplementary Figure 3A–3C).

In addition, we studied the secretion of cytokines in these NKM cells: interleukin (IL)-4, IL-6, IL-10, tumor necrosis factor (TNF)-α, and interferon (INF)-ɣ. The media from 10 cultured NKM cells were collected and analyzed. The concentration of IL-4, IL-6, IL-10 and TNF-α was very low (<100 pg/mL), whereas the INF-ɣ concentration was very high (~300 pg/mL on average) (Supplementary Figure 4).

Taken together, these data suggested that the *in vitro* cytotoxicity of NKM cells was caused mainly by the cytotoxicity of CD56^+^CD16^+^ NK cells, and was suppressed by T_h_ cells.

### Potential function of NKM cells for adjuvant cancer treatment (*in vivo* cytotoxicity)

**Figure 4:**
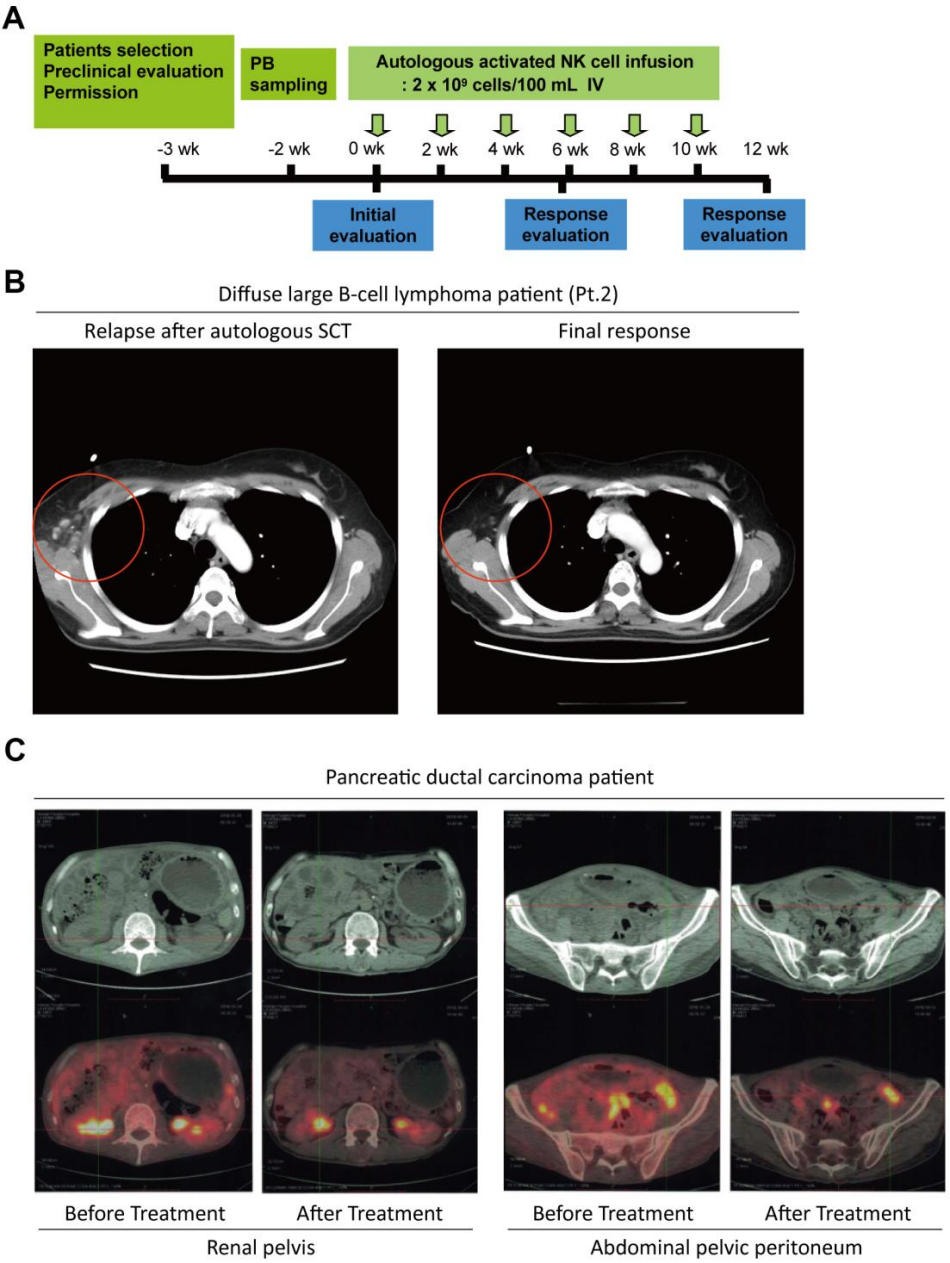
**The potential anti-tumor activity of NKM cells.** (**A**) The schedule of NKM-cell therapy for cancer patients. After physical examination, 20–30 ×10^8^ NKM cells were used for each injection, and six injections were administered in one course of treatment, with a time interval of 2 weeks. (**B**) A complete response from a DLBCL patient (Pt. #2) undergoing NKM-cell treatment. (**C**) A complete response from a pancreatic-cancer patient to NKM-cell treatment (six treatments, once every week). The number of metastatic foci in the renal pelvis and peritoneum of the abdominal pelvis was reduced significantly after NKM-cell treatment.

After the characterization of NKM cells, the potential *in vivo* anti-tumor activities of NKM cells were investigated in cancer patients for adjuvant cancer therapy. We recruited 11 patients with malignant lymphoma (diffuse large B-cell lymphoma [seven patients], peripheral T-cell lymphoma [two patients], anaplastic large-cell lymphoma [one patient], and Hodgkin’s lymphoma [one patient]) who suffered primary relapse/were refractory to treatment (one patient), relapsed after salvage chemotherapy (eight patients), or relapsed after autologous stem-cell therapy (two patients). The treatment process is shown in Figure 4A. Each patient received an intravenous injection of 20–30 ×10^8^ NKM cells each time (treatment once every 2 weeks). The response to NKM treatment was not robust (a complete response in one case and stable disease in one case) (Supplementary Table 2), but quality of life was improved greatly. In the patient who had a complete response (case #2), the number of cancer foci was reduced significantly after treatment with NKM cells (Figure 4B). During and after intravenous infusion of NKM cells, the body temperature, blood pressure, and heartbeat remained stable, and adverse reactions were not found. The patients tolerated the treatment and clinical side-effects were absent.

Systematic reviews of therapy with NK cells have indicated that immunotherapy with allogeneic NK cells has better clinical efficacy than that using autologous cells [29–31]. Thus, we recruited another patient with pancreatic ductal carcinoma (T4N1M1c) whose cancer cells had metastasized, and he could not sustain chemotherapy. Then, we treated this patient with a human leukocyte antigen-matched infusion of haploidentical NKM cells (donor was the patient’s son). The patient received an intravenous injection of 20–30×10^8^ NKM cells every week. After six treatments, the patient showed good toleration of treatment, and the number of metastatic foci in the renal pelvis and abdominal pelvic peritoneum was reduced significantly (Figure 4C). The patient’s vital signs and physical status were improved significantly.

Taken together, these results suggested that NKM cells displayed efficacious anti-tumor activity in cancer patients, and suggested that NKM cells could be used for adjuvant cancer therapy.

### Identification and recharacterization of sub-healthy individuals on the basis of PD-1 expression in PBMCs

**Figure 5:**
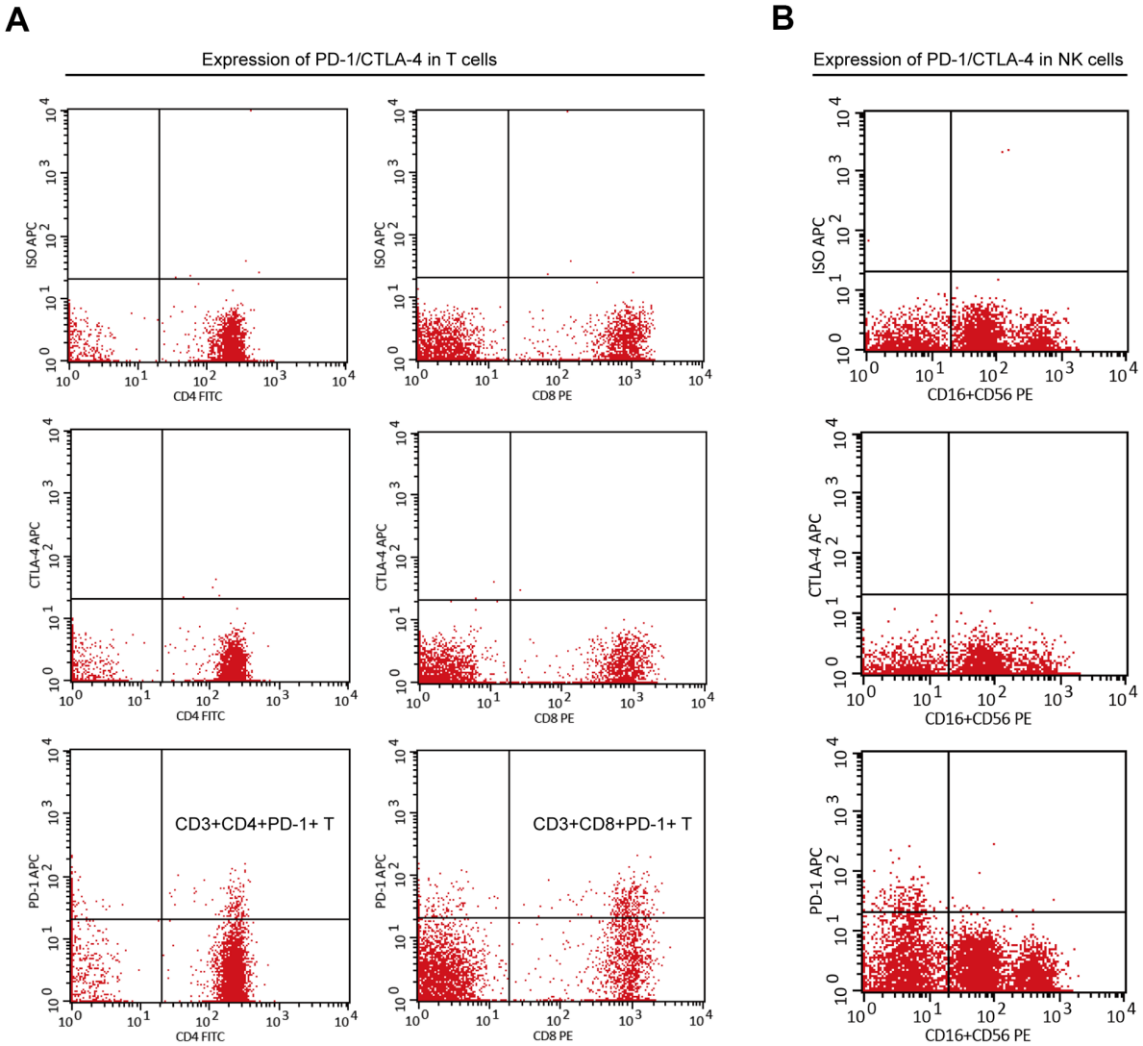
**The expression of PD-1 or CTLA-4 in T cells and NK cells of PBMC.** (**A**) PD-1 was expressed on peripheral T cells (including CD3^+^CD4^+^ T cells and CD3^+^CD8^+^ T cells), but CTLA-4 was not. (**B**) PD-1 and CTLA-4 were not expressed on peripheral NK cells.

When analyzing the PBMCs of cancer patients, researchers found that PD-1 expression was significantly induced in peripheral immune cells [18, 19, 32–34]. Previously, we found that among adolescents (10–20 years), the proportion of PD-1^+^ PBMCs (CD3^+^CD4^+^PD-1^+^ or CD3^+^CD8^+^PD-1^+^) was very low (<2%) (data not shown). However, the proportion of PD-1^+^ PBMCs in adults varies greatly, and the proportion in individuals is stable if external intervention is not applied. Here, to identify sub-healthy individuals, we measured expression of PD-1 and cytotoxic T lymphocyte-associated protein (CTLA)-4 in the NK cells and T cells of PBMCs in 95 individuals aged >50 years (Supplementary Table 3). PD-1 was expressed only on T cells, including T_h_ cells and cytotoxic T cells, but not on NK cells, whereas CTLA-4 was not expressed in any PBMCs (Figure 5A, 5B). High expression of PD-1 in T cells may indicate that a large number of T cells are exhausted [13, 14] which, in turn implies an abnormal immune system.

**Figure 6:**
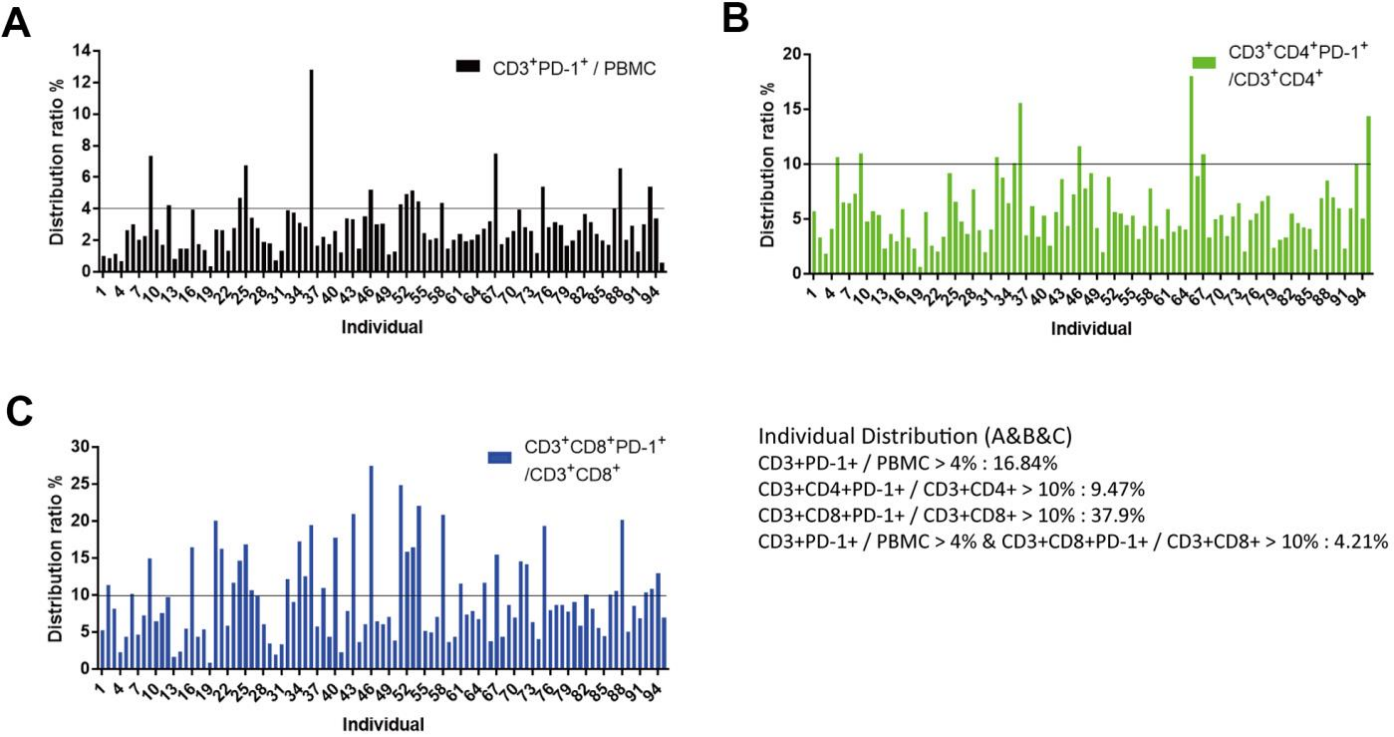
**Identification of “sub-healthy individuals” with PD-1 expression in PBMCs.** (**A**) Proportion of CD3^+^PD-1^+^ cells was <4% in the PBMCs of healthy individuals. PBMCs of 95 individuals (aged >50 years) were analyzed, of which 16.84% of samples had a proportion of CD3^+^PD-1^+^ cells >4%. (**B**) Proportion of CD3^+^CD4^+^PD-1^+^ cells was <10% in the CD3^+^CD4^+^ T cells of healthy individuals. In these 95 individuals, 9.47% of samples had a proportion of CD3^+^CD4^+^PD-1^+^ cells >10%. (**C**) Proportion of CD3^+^CD8^+^PD-1^+^ cells was <10% in the CD3^+^CD8^+^ T cells of healthy individuals. In these 95 individuals, 37.9% of samples had a proportion of CD3^+^CD8^+^PD-1^+^ cells >10%. We defined CD3^+^PD-1^+^ cells/PBMCs >4% and CD3^+^CD8^+^PD-1^+^ cells/CD3^+^CD8^+^ cell >10% as “sub-healthy individuals” (4.21%).

The proportion of CD3^+^PD-1^+^ cells in PBMCs was usually <4%, and only 16.8% of individuals had >4% of CD3^+^PD-1^+^ cells in PBMCs (Figure 6A). The proportion of CD3^+^CD4^+^PD-1^+^ cells in CD3^+^CD4^+^ T cells was usually <10%, and only 9.5% of individuals had >10% of CD3^+^CD4^+^PD-1^+^ cells in CD3^+^CD4^+^ T cells (Figure 6B). The proportion of CD3^+^CD8^+^PD-1^+^ cells in CD3^+^CD8^+^ T cells was usually <10%, and 37.9% of individuals had >10% of CD3^+^CD8^+^PD-1^+^ cells in CD3^+^CD8^+^ T cells (Figure 6C). To define sub-healthy individuals, we set the threshold of the ratio of CD3^+^PD-1^+^ cells/PBMC cells to >4% and the threshold of the ratio of CD3^+^CD8^+^PD-1^+^ cells/CD3^+^CD8^+^ cells to >10%. Furthermore, we measured PD-1 expression in NKM cells. The proportion of CD3^+^PD-1^+^ cells and CD3^+^CD4^+^PD-1^+^ cells was increased in NKM cells, whereas the proportion of CD3^+^CD8^+^PD-1^+^ cells was reduced significantly in NKM cells (p < 0.0001) (Supplementary Figure 5). About 4.2% of individuals in our cohort showed these characteristics, so the immune system of these people may be abnormal, and we defined these people as “sub-healthy individuals”.

### Potential immune normalization of autologous NKM cells in sub-healthy individuals

NKM cells are a mixture of immune-cell types, including T_h_ cells (which inhibit cytotoxicity) and NK cells (which elicit cytotoxicity). We wondered if these NKM cells may display more efficacious functions in the immune normalization of sub-healthy individuals.

**Figure 7:**
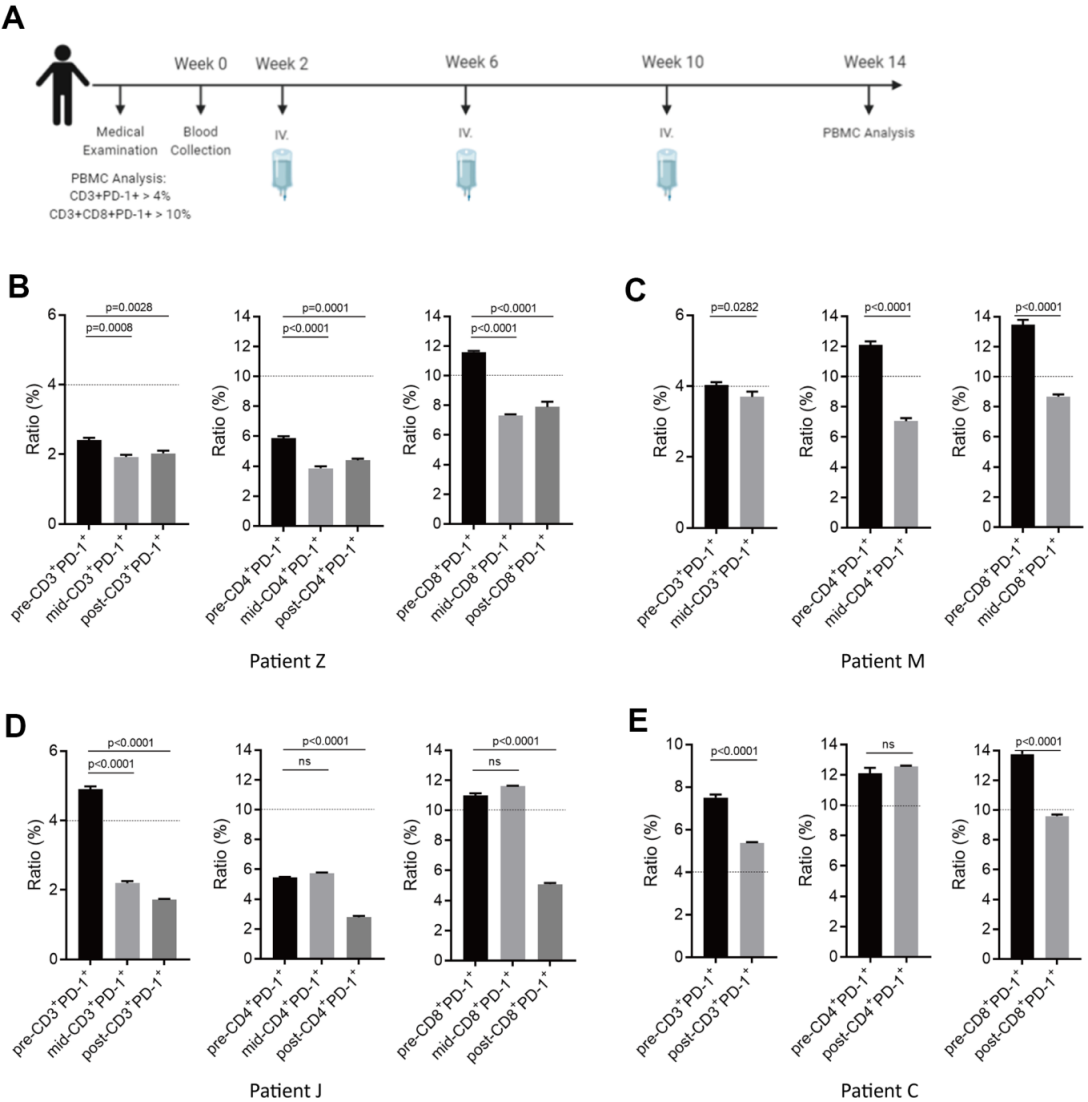
**Potential immune normalization using NKM-cell therapy in sub-healthy individuals.** (**A**) The schedule of NKM-cell therapy for sub-healthy individuals. After physical examination, 20–30 ×10^8^ NKM cells were used for each injection, and three injections were administered in one course of treatment, with a time interval of 1 month. Detection of the therapeutic effect during NKM-cell treatment was undertaken before the second injection. Detection of the final effect was undertaken 1 month after the end of NKM-cell treatment. (**B**–**E**) Efficacious NKM-cell therapy for immune normalization in four sub-healthy individuals. Patient Z (**B**), Patient M (**C**), Patient J (**D**) and Patient C (**E**) were recruited. Proportions of CD3^+^PD-1^+^ cells/PBMCs and CD3^+^CD8^+^PD-1^+^ cells/CD3^+^CD8^+^ cells were measured before (pre-), during (mid-) and after (post-) NKM-cell treatment. The P-values are shown.

We recruited four potential sub-healthy individuals and the treatment schedule is shown as Figure 7A. Approximately 20×10^8^ autologous NKM cells were used for each treatment. Measurement of PD-1 expression in T cells was done before treatment, after the first injection, and after the third injection. In Patient-M and Patient-Z, upon a single injection of NKM cells, PD-1 expression in CD3^+^, CD3^+^CD8^+^, and CD3^+^CD4^+^ cells could be reduced significantly to a normal level (CD3^+^PD-1^+^ cells/CD3^+^ cells was <4%; CD3^+^CD4^+^PD-1^+^ cells/CD3^+^CD4^+^cells was <10%; CD3^+^CD8^+^PD-1^+^ cells/CD3^+^CD8^+^ cells was <10%) (Figure 7B, 7C). In Patient-J, the effect of one injection of NKM cells was not obvious. After three injections of NKM cells, PD-1 expression in CD3^+^, CD3^+^CD4^+^, and CD3^+^CD8^+^ cells decreased eventually to a normal level (Figure 7D). In Patient-C, three injections of NKM cells reduced PD-1 expression in CD3^+^ cells and CD3^+^CD8^+^ cells, but not in CD3^+^CD4^+^ cells (Figure 7E). All sub-healthy individuals showed good tolerance to treatment, and side-effects were not observed.

In summary, we found that with three therapies with autologous NKM cells, we could reduce PD-1 expression in PBMCs, and achieve renormalization of the immune system in sub-healthy individuals.

## DISCUSSION

For decades, researchers have focused efforts on "enhancing/improving" the immune system to eliminate the potential risks of serious diseases (e.g., cancer). The development of immune-cell therapies have made these concepts a reality.

A safe and efficacious method using NKM cells for the potential adjuvant treatment and prevention of cancer is presented here. We characterized the composition of NKM cells and investigated their cytotoxicity *in vitro* and *in vivo*. We showed that NKM cells were significantly cytotoxic to K562 cells or hepatocellular carcinoma cells [35]. However, compared with the number of cancer cells in cancer patients, the number of NKM cells that can reach a cancerous area is relatively small, so the anti-cancer effect is limited. Although the study cohort was and treatment effect was not robust, we showed the potential adjuvant anticancer function of NKM-cell therapy. The latter may not be as efficacious as therapy using pure NK cells. Nevertheless, due to its ease of operation and safety (fewer immune-related side-effects were found), we believe that NKM-cell therapy is potent adjuvant therapy for cancer patients, especially in combination with surgery, to improve the immune response and eliminate residual cancer cells.

Recently, scholars have proposed that cancer immunotherapy should focus on normalization of the immune system [6]. The proportion of PD-1^+^ immune cells (exhausted immune cells) is increased in the cancer tissues and PBMCs of cancer patients [13, 20, 23]. We postulate that this abnormality in PD-1 expression in PBMCs may be related to a high risk of cancer. A high proportion of PD-1^+^ T cells in PBMCs was found in some older adults (4.21%) in our cohort. Among the sub-healthy individuals defined in our research, NKM-cell therapy could reduce PD-1 expression in PBMCs and quickly restore/rebalance the immune system. However, for some individuals (e.g., Patient C), more injections or larger doses of NKM-cell therapy may be required to reduce PD-1 expression in PBMCs.

We expanded NKM-cell therapy to not only treat cancer patients, but also prevent cancer in patients through normalization of the peripheral immune system. NKM-cell therapy for sub-healthy individuals must be studied in randomized clinical trials. Our study could be used as reference to expand the number of applications of immune-cell therapy.

The COVID-19 pandemic is wreaking havoc on healthcare and economic systems worldwide. In patients with severe COVID-19, reduction in the number of immune cells and exhaustion of functional T cells in peripheral blood has been documented [28]. Just like our sub-healthy individuals, PD-1 expression is increased significantly in the CD4^+^ T cells and CD8^+^ T cells of PBMCs in COVID-19 patients. Our experiments in sub-healthy individuals normalized their peripheral immune system, so NKM-cell treatment for COVID-19 patients could be possible.

## CONCLUSIONS

We demonstrated that a new method using NKM cells was safe and efficacious as adjuvant treatment for cancer patients as well as therapy for sub-healthy individuals. Normalization of the peripheral immune system through NKM-cell therapy could expand its scope of application in different disorders.

## MATERIALS AND METHODS

### Culture of NKM cells

NKM cells were manufactured in an ISO Class 5 Good Manufacturing Practice Laboratory within the International Research Center for Regenerative Medicine in BOAO International Hospital (Qionghai, China). Primary PBMCs were separated from whole blood (30–60 mL). A T75 flask was pre-coated with OKM25 medium (Fukoku, Ageo, Japan). PBMCs from individuals were cultured for 3–5 days in T75 and T225 flasks with OKM100 medium containing 1750 JRU/mL of IL-2 (Fukoku) and 10% autologous inactivated plasma. Then, NKM cells were transferred to a CO_2_ Bag containing 1 L of OKM200 medium (containing 175 JRU/mL of IL-2) (Fukoku) and 1% autologous inactivated plasma, followed by culture for 3–5 days. NKM cells were incubated at 37° C in a humidified incubator in an atmosphere of and 5% CO_2_.

### Ethical approval and consent to participate

The study protocol was approved by the Medical Ethics Committee of BOAO International Hospital. Sample collection, written informed consent from and recruitment of healthy donors and patients followed Ethics Review Board protocols from BOAO International Hospital (QiongHai, China; approval number of BIH-2018-1001 for adjuvant cancer therapy, and BIH-2018-1002 for care of sub-healthy individuals). The study was conducted according to the principles of the Declaration of Helsinki 1964 and its later amendments.

### Flow cytometry and antibodies

We wished to analyze the T-cell subpopulations of PBMCs and NKM cells. BD Tritest™ CD3 fluorescein isothiocyanate (FITC)/CD16^+^CD56 phycoerythrin (PE)/CD45 peridinin chlorophyll protein (PerCP) and BD Tritest CD4 FITC/CD8 PE/CD3 PerCP (BD Biosciences, San Jose, CA, USA) were used for flow cytometry. BD Pharmingen™ PE Mouse Anti-Human CD56 and BD Pharmingen FITC Mouse Anti-Human CD16 (BD Biosciences) antibodies were used for analyses of the NK cell-subpopulations of PBMCs and NKM cells. BD Pharmingen APC Mouse anti-Human CD279, BD Pharmingen APC Mouse Anti-Human CD152, and BD Pharmingen APC Mouse IgG1, κ Isotype Control antibodies (BD Biosciences, Shanghai, China) were used for flow cytometry of the PD-1^+^ cell and CTLA-4^+^ cell subpopulations of PBMCs and NKM cells. Flow cytometry was done according to manufacturer (BD Biosciences) instructions.

### Assay to measure cell cytotoxicity

The cytotoxicity of PBMCs and NKM cells was assessed by measuring the mean fluorescence intensity of Calcein AM (catalog number, C3099; Thermo Fisher Scientific, Waltham, MA, USA) in intact cancer cells using Terascan VPC (Minerva Tech, Tokyo, Japan). Briefly, target cells (cancer cell line K562) were resuspended with Dulbecco’s modified Eagle’s medium containing 10% fetal bovine serum, and mixed with 20 μL of Calcein AM at 37° C for 30 min for staining. PBMCs or NKM cells were co-cultured with pre-stained target cells at a particular E:T ratio for 2 h or 4 h. The mean fluorescence intensity of living target cells was measured. The percent cytotoxicity of cells was calculated using the following formula [35]: % cytotoxicity = (1 − [(average fluorescence of the sample wells − average fluorescence of the maximal-release control wells)/(average fluorescence of the minimal-release control wells − average fluorescence of the maximal-release control wells)]) ×100.

### Sorting of immunological magnetic beads

Cell subsets of NKM cells were sorted using an NK Cell Isolation Kit (130-092-657) for NK cells, CD3 MicroBeads (130-050-101) for CD3^+^ T cells, CD4 MicroBeads (130-045-101) for CD4^+^ T cells, and CD8 MicroBeads (130-045-201) for CD8^+^ T cells according to manufacturer (Miltenyi Biotec, Bergisch Gladbach, Germany) instructions. Isolated cells (negative and positive) were sorted further with a flow cytometer.

### Cytokine analyses with a cytometric bead array (CBA) kit

The cytokines secreted by NKM cells were analyzed by Human Th1/Th2 Cytokine Kit II (551809) from BD Biosciences. NKM cells (~2×10^9^ cells) were harvested from 1.5 L of culture medium. The cytokines in these media were dyed and analyzed by flow cytometry according to the instructions of the CBA-kit supplier. Then, the collected data were analyzed with FCAP Array v3.0 (BD Biosciences).

### Statistical analyses

Experiments were carried out independently in triplicate. Data are the mean ± SD. Graphs were plotted and analyzed with Prism 7.1 (GraphPad, La Jolla, CA, USA). P < 0.05 was considered significant.

### Availability of data and material

All data have been added in the Supplementary Tables.

## Supplementary Material

Supplementary Figures

Supplementary Table 1

Supplementary Table 2

Supplementary Table 3
