# Supplementary material for: Immune normalization strategy against suboptimal health status: safe and efficacious therapy using mixed-natural killer cells
Source: Aging (Albany NY). 2021 Aug 30;13(16):20131–48. doi: 10.18632/aging.203279 (PMC8436936; doi:10.18632/aging.203279)
Supplement: Supplementary Figures [file aging-13-203279-s001.pdf]

SUPPLEMENTARY FIGURES

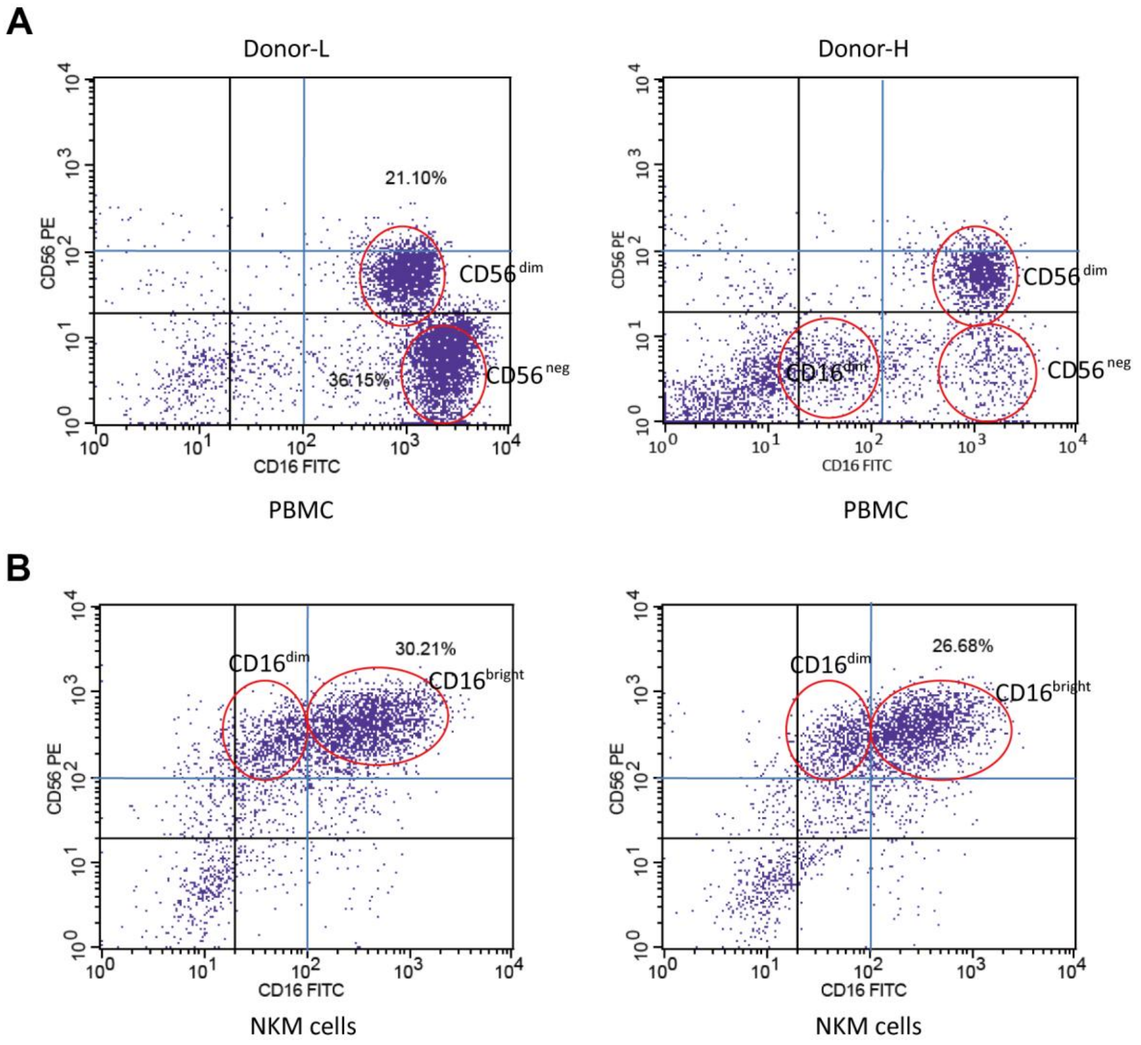

Supplementary Figure 1. The subpopulation of NK cells in PBMC and NKM. (**A**) The majority of NK cells in PBMC was CD16^{bright} CD56$^{dim/neg}$ NK cells. In the PBMC of donor-N, NK cells were composed of two main subpopulations: CD16^{bright}CD56^{dim} NK cells and CD16^{bright}CD56^{neg} NK cells. In the PBMC of donor-H, NK cells were composed of three main subpopulations: CD16^{bright}CD56^{dim} NK cells, CD16^{bright}CD56^{neg} NK cells and CD16^{dim}CD56^{neg} NK cells. (**B**) The majority of NK cells in NKM was CD16^{bright}CD56^{bright} NK cells. In the NKM, NK cells were composed of two main subpopulations: CD16^{bright}CD56^{birght} NK cells and CD16^{dim}CD56^{birght} NK cells.

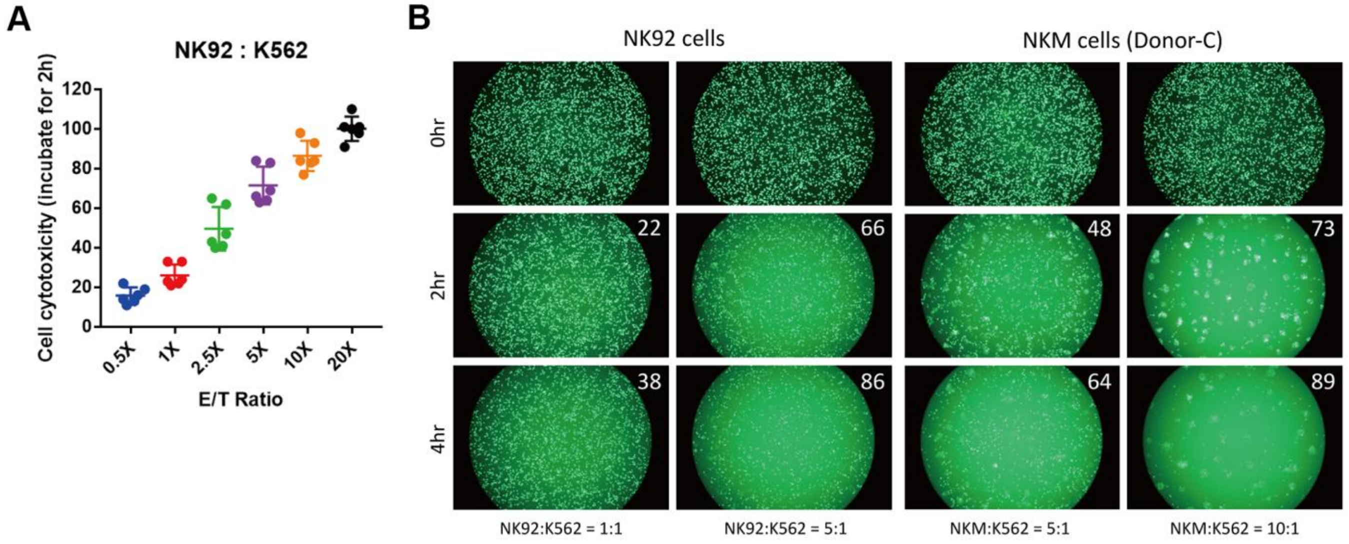

Supplementary Figure 2. The comparison of cytotoxicity between NKM and NK92 cells. (**A**) Different E/T ratio showed high cytotoxicity of NK92 cells. (**B**) Labeled target cells (K562 cancer cells) were killed by the effector cells (NKM or NK92). The number on the upper right showed the cytotoxicity of the effector cells. The ratio of effector cells and target cells was 1:1 or 5:1 or 10:1, and the incubation time was 2h or 4h.

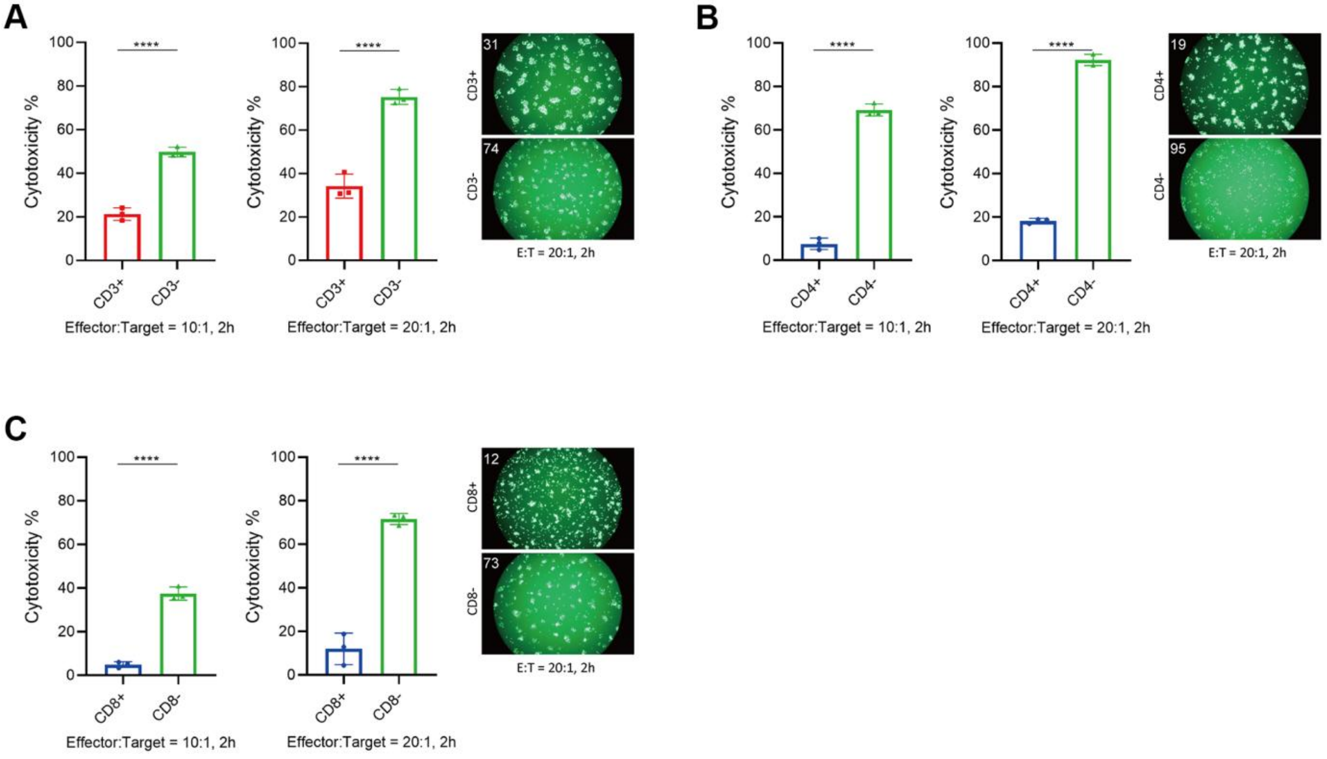

Supplementary Figure 3. The cytotoxicity of CD3+ vs CD3- cells, CD4+ vs CD4- cells, CD8+ vs CD8- cells. (**A**) The NKM cells were separated into CD3+ ($CD3^+$ cells) and CD3- (other non-CD3 cells) cells with immunomagnetic beads (CD3 MicroBeads). (**B**) The NKM cells were separated into CD4+ ($CD4^+$ cells) and CD4- (other non-CD4 cells) cells with immunomagnetic beads (CD4 MicroBeads). (**C**) The NKM cells were separated into CD8+ ($CD8^+$ cells) and CD8- (other non-CD8 cells) cells with immunomagnetic beads (CD8 MicroBeads). The ratio of effector cells: target cells was 10:1 or 20:1, and the incubation time was 2 h. The number on the upper right shows the cytotoxicity of effector cells.

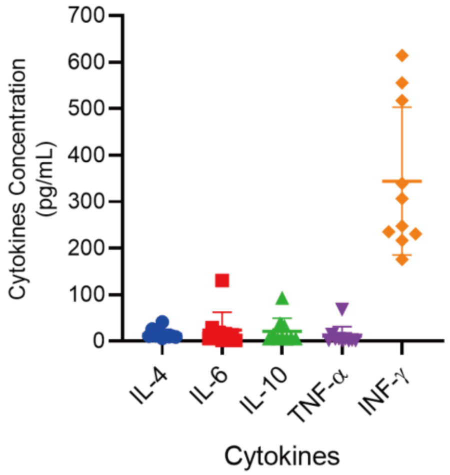

Supplementary Figure 4. The secretion of cytokines in NKM cells. The cytokines: interleukin (IL)-4, IL-6, IL-10, tumor necrosis factor (TNF)-α, and interferon (INF)-γ were investigated. The media from 10 cultured NKM cells were collected and analyzed.

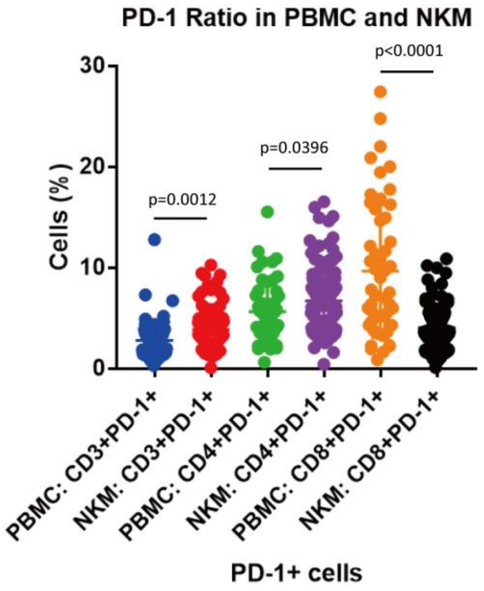

Supplementary Figure 5. The PD-1 expression rate in the subpopulations of NKM cells. The ratio of PD-1 expressing CD3 T cells, CD4 T cells, and CD8 T cells were calculated in our NKM cells.
