# Supplementary material for: Immune normalization strategy against suboptimal health status: safe and efficacious therapy using mixed-natural killer cells
Source: Aging (Albany NY). 2021 Aug 30;13(16):20131–48. doi: 10.18632/aging.203279 (PMC8436936; doi:10.18632/aging.203279)
Supplement: Supplementary Table 2 [file aging-13-203279-s003.pdf]

Supplementary Table 2. Safety and efficacy of NKM treatment in malignant lymphoma.

Patient	Age/sex	Malignant lymphoma (subtype)	LDH	No. of salvage	IPI	Bulky	B symptom	No. of infusion	Mid response	Final response	Withdrawal
Pt. 1	61/M	DLBCL	221	8	3	No	No	5	SD	SD	
Pt. 2	38/F	DLBCL	177	7+auto	1	No	No	6	PR	CR	
Pt. 3	67/M	DLBCL	293	8	3	No	Yes	5	PD	PD	
Pt. 4	63/F	DLBCL	398	3	4	No	No	3	PD	PD	Progression
Pt. 5	64/M	DLBCL	196	2	3	No	No	2	PD		Progression
Pt. 6	55/F	DLBCL	1506	8	4	No	No	3	PD		Progression
Pt. 7	46/F	DLBCL	519	8+RIT	2	Yes	No	6	PD	PD	
Pt. 8	41/M	PTCL/enteropathic T cell	202	8+auto	3	No	No	3	PD		
Pt. 9	46/M	PTCL/enteropathic T cell	267	2	4	No	No	1	NM		expire
Pt. 10	52/M	Anaplastic large cell	625	4	2	Yes	No	3	PD		Progression
Pt. 11	45/M	Hodgkin's lymphoma	155	0	2	No	No	3	SD		
